# Elastic Properties of Jute Fiber Reinforced Polymer Composites with Different Hierarchical Structures

**DOI:** 10.3390/ma15197032

**Published:** 2022-10-10

**Authors:** Phani Prasanthi, Sivaji Babu Kondapalli, Niranjan Kumar Sita Rama Morampudi, Venkata Venu Madhav Vallabhaneni, Kuldeep Kumar Saxena, Kahtan Adnan Mohammed, Emanoil Linul, Chander Prakash, Dharam Buddhi

**Affiliations:** 1 Department of Mechanical Engineering, Prasad V. Potluri Siddhartha Institute of Technology, Kanuru, Vijayawada 520007, Andhra Pradesh, India; 2 Department of Mechanical Engineering, V. R. Siddhartha Engineering College, Kanuru, Vijayawada 520007, Andhra Pradesh, India; 3 Department of Mechanical Engineering, GLA University, Mathura 281406, Uttar Pradesh, India; 4 Department of Medical Physics, Hilla University College, Babylon 51002, Iraq; 5 Department of Mechanics and Strength of Materials, Politehnica University Timisoara, 1 Mihai Viteazu Avenue, 300222 Timisoara, Romania; 6 School of Mechanical Engineering, Lovely Professional University, Phagwara 144411, Punjab, India; 7 Division of Research and Development, Lovely Professional University, Phagwara 144011, Punjab, India; 8 Division of Research & Innovation, Uttaranchal University, Dehradun 248007, Uttarakhand, India

**Keywords:** jute fiber reinforced composites, hierarchical structures, micromechanics, finite element method, elastic properties

## Abstract

A two-stage micromechanics technique is used to predict the elastic modulus, as well as the major and minor Poisson’s ratio of unidirectional natural fiber (NF) reinforced composites. The actual NF microstructure consists of cellulose, hemicellulose, lignin, lumen, etc., and these constituents and their contributions are neglected in classical models while quantifying their mechanical properties. The present paper addresses the effect of the real microstructure of the natural jute fiber (JF) by applying a micromechanics approach with the Finite Element Method. Six different hierarchically micro-structured JFs are considered to quantify the JF elastic properties in the first level of homogenization. Later, the JF reinforced polypropylene matrix properties are investigated in the second stage by adopting a homogenization approach. Taking into account the different hierarchical structures (HS), the fiber direction modulus (E_1_), transverse modulus (E_2_ and E_3_), in-plane and out-of-plane shear modulus (G_12_ and G_23_), and major (ν_12_, ν_13_) and minor (ν_23_, ν_21_) Poisson’s ratios are estimated for JF and JF reinforced polypropylene composites. The predicted elastic modulus from micromechanics models is validated against the analytical results and experimental predictions. From the present work, it is observed that the HS of NF needs to be considered while addressing the elastic properties of the NF-reinforced composite for their effective design, particularly at a higher volume fraction of NF.

## 1. Introduction

Natural fibers (NFs) are also called lignocellulosic fibers, which are extracted from plants, and these fibers contain different proportions of cellulose, hemicelluloses, lignin, and lumen. The main constituent among all of these is cellulose, and the percentage of cellulose is in the range of 50–70% [1]. Microfibrils are formed from these cellulose chains, and these microfibrils aggregate together to form macrofibrils through an amorphous matrix termed lignin and hemicellulose. The hollowness in this structure is called the lumen. Cellulose, hemicellulose, lignin, and lumen are the main constituents of plant-based fibers. Based on the differences in the percentage of contributions in the NF, the hierarchical structure (HS) may differ. The use of lignocellulosic fibers has great benefits in terms of biodegradability, energy-friendly production, and has the potential to replace synthetic fibers [2,3].

Due to their mechanical strength and stiffness as well as thermal and tribological properties, jute, bamboo, sisal, flax, kenaf, and hemp fibers are recommended as NFs for the manufacture of natural fiber reinforced (NFR) composites [4]. With the increasing application of NFR composites in the automotive, aerospace, marine, sporting goods, biomedical, and electronic industries, the design and development of NFR composites is becoming a challenging task [5,6]. Because of the difficulties associated with their fabrication and experimentation, many researchers turn to computational methods for the mechanical and thermal characterization of NFR composites [7,8]. The finite element (FE) method is the most commonly used tool in the modelling of NFs and NFR composites to predict the elastic and thermal properties. Due to the differences in the microstructure of plant-based fibers, the analysis of NFs is a challenging task [9]. Using multi-scale homogenization computational methods, the mechanical relationships of a large-scale fiber-reinforced composite material can be established from a small-scale fiber [10]. Using multi-scale FE analysis, the natural flax fiber reinforced composite properties of lamina and laminate are estimated through representative volume elements (RVEs). Using the inputs from the micro-scale (lamina) analysis, the macro-scale (laminate) analysis was performed, and the tensile strength and flexural behavior were estimated [11]. Also, using FE-based software, the acoustic behavior of sisal fiber reinforced composite is estimated by modelling three-layer fiber structures with technical fiber and microfibrils. In this case, the failure process and stress distribution are estimated from the FE models [12]. Two-stage homogenization processes are adopted to predict the elastic properties of NF. The effect of the lumen ratio of NF on the axial Young’s modulus of NF was analyzed, and it was concluded that the prediction of the axial Young’s modulus of NF would be in good agreement with the experimental data by knowing the exact lumen ratio [13]. Although NFs show similar morphology, the differences in the internal area of the lumens and the number of lumens make them different from each other. In addition, the combined effect of chemical and morphological composition on the tensile behavior of fibers is investigated [14]. The elastic modulus of Kevlar 29 and Kevlar 49 fibers was calculated using multi-scale modelling techniques [15]. Using multi-scale homogenization techniques and RVE models, the elastic properties of the dry sugarcane leaf reinforced polymer composite are estimated by adopting ANSYS software. To estimate the elastic properties of dry sugarcane leaf-reinforced epoxy composite using micromechanics analysis, dried sugarcane leaves are considered as rectangular inclusions [16].

Biodegradable cellulose-based fiber-reinforced composite materials’ behavior under low velocity is estimated and compared to the experimental results. Knowing this impact strength, the FE method can be used to provide a new application for biodegradable composite materials [17]. From the above works, it can be seen that the FE method and micromechanics can be used to model and analyze cellulose if homogenization techniques are used [15,18,19]. The RVE method is the most efficient homogenization-based multi-scale model when applied to cellulose-based composites, and represents the relevant features of NF in the uniform structure [20]. In terms of elastic and thermal properties, NFs and matrix constituents have a mismatch effect. The mismatch in the properties has a clear effect on the interfacial shear strength. The mismatch effect on the properties can also be estimated by adopting a micromechanics approach [21,22]. Many authors have used two-stage homogenization techniques to characterize the elastic and thermal behavior of composite materials in the presence of defects such as voids and debonding [23,24]. NFR composites’ mechanical properties are certainly influenced by non-cellulosic compounds such as hemicelluloses, lignin, waxes, pectin, etc. However, it is possible to decrease the percentages of non-cellulosic compound by opting for an appropriate fiber treatment process [25,26]. Estimates of NF properties are unclear with respect to testing, i.e., experiments performed on individual fibers or fiber bundles. This information is not clearly explored. If findings are based on a single fiber, the hollowness of the lumen is considered [27], and the fraction of cross-sectional area taken up by the lumen has been found to be 27.2%, 6.8%, and 34.0% for sisal, flax, and jute, respectively. It was noted that the presence of lignin makes the cellulose rigid [28]. These jute fiber reinforced (JFR) composites have a wide range of applications in household, engineering, building structures, door frames, furniture, shopping bags, etc. and these composites can be thermally stable in the range of 250–365 °C [29]. Another important aspect of jute fiber (JF) is the large lumen, which needs to be reflected in the manufacturing process of composite materials. This hollow lumen has a clear influence on the stiffness of the NF composites. These lumen percentages must be considered when designing the properties of NF composites [30]. JFs consist of a high percentage of hollow lumen structures, which could be beneficial for sound energy conversion [31]. However, this lumen will never contribute to the mechanical properties [32] and will remain as it is inside the composites, and the lumen is a large tubular void in the middle of each NF [33,34].

From the above findings, the authors of this paper observed that the identification of lumen and cellulose percentages is the key step in tailoring the properties of NF composites. The lumen represents the hollowness of the NF and the cellulose promotes the strength of the fiber. Most NF studies have been limited to E, i.e., Young’s modulus in the longitudinal direction. However, NFR composites are orthotropic in nature, requiring the use of nine elastic constants for effective design of NFR composite structures. This data is not yet available for JFR composites. There are few studies on Poisson’s ratio, with more emphasis on elastic modulus and shear modulus. However, Poisson’s ratio requires knowledge of the coupling between deformation in the lateral and longitudinal directions of composite materials. While Poisson’s ratio does not matter much for regular materials, it does matter a lot for composite materials, which are made of two different materials that work together when loaded. This behavior can be identified by Poisson’s ratio. At the same time, the fiber alignment direction also plays a definite role in Poisson’s ratio and must be taken into account. Lumen hollowness and its percentage are dependent on the type of NF. This lumen percentage influence on all elastic properties has not been addressed so far. While comparing the experimental and analytical or simulation studies of the properties of NF composites, the experimental results consider all NF constituents, i.e., cellulose, hemicelluloses, lignin, and lumen, while in the analytical or simulation studies, only the technical fiber [12] property will be considered to estimate the overall composite properties. Considering the aforementioned knowledge gaps, the present problem focuses on the estimation of the nine elastic properties of a jute NFR composite considering the six HSs with different lumen percentages using analytical and simulation studies.

## 2. Material and Methods

Experimental procedures for estimating the longitudinal modulus of straight natural fiber reinforced composite and their mechanical characterization are well described in the previous article [35]. Using the same procedure, the JFR epoxy composites are prepared and tested. The JFs are treated with NAOH solution and the weight of straight JFs is measured and placed in a mold. The epoxy matrix is poured over the JF according to the fiber weight fraction. The mold is cured for 24 h after which the specimen is removed from the mold. Subsequently, the specimens are cut from the same lamina according to ASTM standards. Five samples are prepared at each weight and tested for longitudinal modulus.

**Figure 1:**
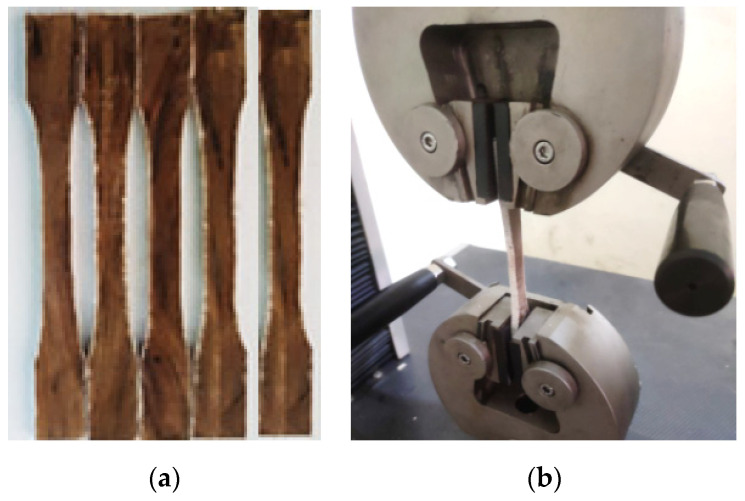
JFR composites specimens at 30% of weight (**a**) and testing of the JFR composite on tensile testing machine (**b**).

Unidirectional JF is used for the fabrication of NFR polymer composites. Epoxy resin (LY556) and compatible hardener (HY951) are used as a hosting medium. Numerical studies are performed considering the volume fraction, and for conducting the experimental studies, the volume fraction is converted to weight fraction using the density of JFs and epoxy resin. The weight fraction of JF is kept at 12.95% and 36.47% based on the volume fraction of JFs (10% and 30%). Composite specimens are prepared using hand layup technique. Five specimens are prepared for each configuration and tested according to the ASTM D638 standard (Figure 1a). The tensile tests were carried out using the Universal Tensile Testing Machine at the Prasad V. Potluri Siddhartha Institute of Technology, Kanuru, Vijayawada, Andhra Pradesh, India (Figure 1b). Table 1 shows the elastic modulus obtained from experimental tensile tests.

## 3. Analytical Studies of Jute Fiber Reinforced Composites

In the plant-based NFs, the strong network of hydrogen bonds between the hydroxyl groups of neighboring chains causes the cellulose to organize in a hierarchical way [36]. Cellulose is the main structural component of plant cell walls [37]. In this work, six types of such structures are taken for analysis and the elastic modulus is estimated. The hierarchical structures (HSs) and the percentage of each constituent are presented in Table 2. 

These HS structures are designed based on the cellulose percentage i.e., some fibers have maximum cellulose (61%) and some fibers have minimum cellulose (39%). Application of the Micromechanics method to the composite materials to evaluate their elastic properties will start with the selection of the Representative Volume Element (RVE). The NFs in the matrix phase are thought to be straight and spread out evenly.

**Figure 2:**
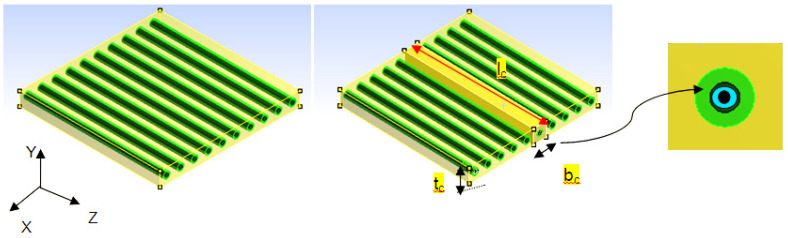
Idealization of natural fiber and selection of RVE with reference to the global coordinate system (X, Y and Z).

The space between the JFs and fiber is b_f_ and the thickness is t_f_. The size of the RVE is represented by l_c_, b_c_ and t_c_ where l_c_ is the length of the RVE, b_c_ is the width of the RVE, and t_c_ is the thickness of the RVE (Figure 2). The RVE shows the whole lamina of the JF, which can be made by putting the RVEs next to each other over and over again. 

### 3.1. Estimation of Elastic Properties of Selected RVE

The selected RVE is an orthotropic body characterized by nine elastic constants; these are longitudinal modulus (E_1_), transverse modulus (E_2_ and E_3_), Major Poisson’s ratio (ν_12_, ν_13_), Minor Poisson’s ratio (ν_21_, ν_23_), in-plane and out-of-plane shear modulus (G_12_, G_23_, G_13_).

#### 3.1.1. Longitudinal Modulus E_1_

To find out the fiber direction modulus of a JFR polymer matrix composite, an electrical analogy was made. When applying the numerical calculations, the HS of JF is taken into account. The HS of NF includes lumen, lignin, hemicellulose, and cellulose. A lumen in the NF is treated as a hollow member; lignin, hemicellulose, and cellulose are different in terms of geometry and material properties. These fibers are uniformly distributed throughout the matrix material.

**Figure 3:**
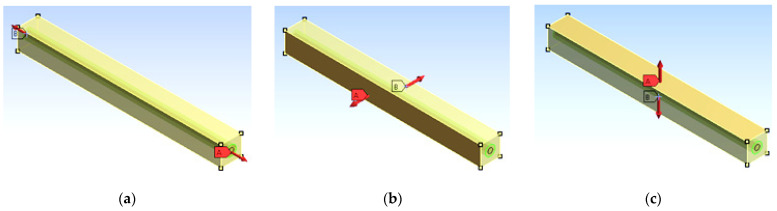
RVE under longitudinal (**a**) and transverse (**b**,**c**) loading. (Red arrows represent the loading direction).

All constituents present share the load acting on the RVE. Lumen, lignin, hemicellulose, and cellulose will take the load as shown in Figure 3a.

The forces shared by all the constituents are given in Equation (1). Using the relation between the forces and stresses, Equation (2) is developed.

(1) FΦ=Fα+Fβ+F γ+Fδ+Fm

(2) σΦ·AΦ=σα×Aα+σβ×Aβ+σγ×Aγ+σδ×Aδ+σm×Am

Using Hooke’s law, the stress is directly proportional to the strain, as follows:

(3) σ=E·ε

Substituting the Equation (3) in (2) gives:

(4) E1Φ·ε1Φ·AΦ=E1α·ε1α·Aα+E1β·ε1β·Aβ+E1γ·ε1γ·Aγ+Eδ·εδ·Aδ+Em·εm ·Am 

Under the condition of a perfect bond between the constituents of the fiber and the matrix, the strain generated in the RVE is equal to the strain in the fiber and the strain developed in the matrix.

(5) ε1Φ=ε1α=ε1β=ε1γ=εδ=εm

The Equation (4) becomes:

(6) $$E1\Phi =E1\alpha \cdot \frac{A\alpha }{A\Phi }+E1\beta \cdot \frac{A\beta }{A\Phi }+E1\gamma \cdot \frac{A\gamma }{A\Phi }+E1\delta \cdot \frac{A\delta }{A\Phi }+\mathrm{Em}\cdot \frac{\mathrm{Am}}{A\Phi \;}$$

(7) E1Φ=E1α·Vα+E1β·Vβ+E1γ·Vγ+E1δ·Vδ+Em·Vm

#### 3.1.2. Transverse Modulus

This modulus is obtained from the RVE subjected to transverse loading as shown in Figure 3b,c.

The transverse elongation under the applied load is equal to the transverse extension generated in all constituents, such as fiber and matrix. Again, JF is considered with lumen, lignin, hemicellulose, and cellulose, considering all the constituents the total elongation is represented as in Equation (8) thus:

(8) ΔΦ=Δα+Δβ+Δγ+∇δ+∇m

Replacing the deformation with the strain, the Equation (9) can be obtained. Using the strain in the above is modified as:

(9) ε2Φ·wΦ=ε2α·wα+ε2β·wβ+ε2γ·wγ+ε2δ·wδ+εm·wm

The transverse strain is obtained by rearranging the equation, the Equation (10) is obtained. Finally the transverse strain in terms of strain of the each constituent and volume fraction of respective constituent the Equation (11) is obtained.

To get the ε2Φ

(10) $$\varepsilon 2\Phi =\varepsilon 2\alpha \cdot \frac{w\alpha }{w\Phi }+\varepsilon 2\beta \cdot \frac{w\beta }{w\Phi }+\varepsilon 2\gamma \cdot \frac{w\gamma }{w\Phi }+\varepsilon 2\delta \cdot \frac{w\delta }{w\Phi }+\varepsilon m\cdot \frac{\mathrm{wm}}{w\Phi }$$

(11) ε2Φ=ε2α·Vα+ε2β·Vβ+ε2γ·Vγ+ε2δ·Vδ+εm·Vm

Using the relation between the strain and stress in terms of modulus in the respective directions, the Equation (11) becomes:

(12) $$\frac{\sigma 2\Phi }{E2\Phi }=\frac{\sigma 2\alpha }{E2\alpha }\cdot V\alpha +\frac{\sigma 2\beta }{E2\beta }\cdot V\beta +\frac{\sigma 2\gamma }{E2\gamma }\cdot V\gamma +\frac{\sigma 2\delta }{E2\delta }\cdot V\delta +\frac{\sigma m}{\mathrm{Em}}\cdot \mathrm{Vm}$$

(13) σ2Φ=σ2α=σ2β=σ2γ=σ2δ=σm

After applying the assumption of Equation (13), the Equation (12) becomes:

(14) $$\frac{1}{E2\Phi }=\frac{V\alpha }{E2\alpha }+\frac{V\beta }{E2\beta }+\frac{V\gamma }{E2\gamma }+\frac{V\delta }{E2\delta }+\frac{\mathrm{Vm}}{\mathrm{Em}}$$

The same analogy is applied to calculate the G_12_ as presented in Equation (15).

(15) $$\frac{1}{G12\Phi }=\frac{V\alpha }{G12\alpha }+\frac{V\beta }{G12\beta }+\frac{V\gamma }{G12\gamma }+\frac{V\delta }{G12\delta }+\frac{\mathrm{Vm}}{\mathrm{Gm}}$$

Substituting the corresponding values of the fiber constituents’ matrix elastic modulus and their percentage in Equations (7), (14) and (15), the longitudinal modulus and transverse modulus and shear modulus of the JFR composite will be estimated, respectively.

## 4. Simulation Studies of Jute Fiber Using Micromechanics Approach

### 4.1. First Stage of Homogenization

Further, using the micromechanics and finite element method, the nine elastic properties of the JFR composites were estimated. The work is carried out in two stages. In the first stage, only JF properties were determined by considering different HSs. Each HS contains different constituents such as cellulose, hemicelluloses, lignin, and lumen in different proportions. This stage is considered the first stage of homogenization. In the second step, the properties of the JFR polypropylene composite are estimated using the Finite Element Based Software ANSYS 19.2. To ensure that the simulation models are accurate, the results of the FE models are checked against the analytical results.

**Figure 4:**
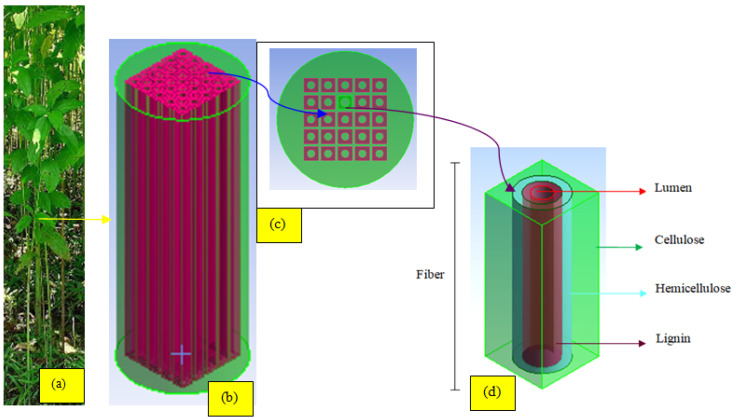
Representation of Natural fiber structure: jute plant (**a**) fiber bundle of the plant (**b**), cross-sectional view of the fiber bundle (**c**) and representation of single fiber with constituents (**d**).

Figure 4a shows the cross section of a unidirectional NFR composite, which is illustrated to understand the HS. A fiber bundle can be seen in Figure 4b and the uniform distribution of each fiber in the fiber bundle is idealized to be spread regularly, and the analysis of one fiber is enough to estimate the fiber bundle properties in Figure 4c,d.

The unit cell contains lumen, cellulose, and matrix phase, which are obtained by selecting a fiber from the bundle. In this case study, it is divided into two phases. In the first stage of homogenization, the JF properties are estimated by including cellulose, lumen, lignin, hemicelluloses, and later, using the properties of the JF with all its constituents, the fiber reinforced matrix properties are estimated. These homogenization concepts are used to understand the potential of electrical systems [38]. Similarly, a transverse thermal conductivity model was recently proposed [39] considering the hollow portion of the NF (lumen), and the remaining portion of the fiber is treated as cellulose. In this work, along with the lumen percentage, the lignin, cellulose, and hemicellulose percentages are also reflected in the RVE to estimate the natural properties of JF. The JF contains between 61–71% cellulose, a large amount of hemicelluloses (14–20%), lignin (12–13%), and pectin (0.2%), as cited in Ref. [40]. The Young’s modulus of each constituent of the fiber is provided in Table 3.

**Figure 5:**
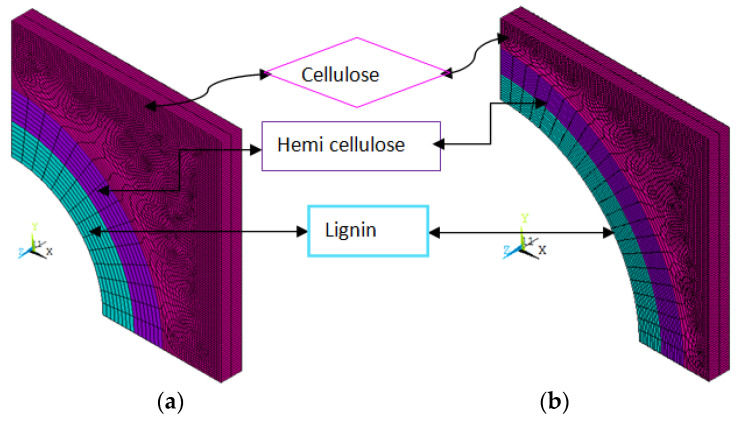
Finite Element Model of H1 (**a**) and H6 (**b**) models.

The properties of the RVE can be estimated by making the RVEs in a square array look like they are perfect and setting the appropriate boundary conditions. The size of the RVE is determined based on the volume fraction of the fiber constituents. For the HS-1 model, the cellulose percentage is 61%, the hemicellulose percentage is 14%, the lignin is 12%, and the lumen is 13%. Based on these percentages, the radius of each constituent is calculated. For this structure, the square RVE size is 10 × 10 nm^2^, and the diameter of the lumen is calculated by equating the percentage of lumen to the size of the RVE, which is the area of the lumen. The lumen is treated as a hollow circle in the square RVE. The radius of the lumen is calculated according to the volume fraction. For example, the volume fraction of lumen in the total volume of the RVE is 13% for the HS-1 model. However, the cross-sectional areas are important in this calculation. The thicknesses of all the constituents are the same in the RVE. Hence, the areas of the constituents represent the volume fractions of the constituents. For the fixed RVE size (10 × 10 nm^2^) and fixed lumen percentage (13% for the HS-1 model), the radius of the lumen is calculated by dividing the lumen area (π/4·d_lu_^2^) to the total RVE area (10 × 10 nm^2^) and equating the outcome to 13% (lumen percentage for the HS-1 model) where d_lu_ is the diameter of the lumen. Similarly, the remaining constituents’ dimensions are also estimated. The cellulose area is obtained by subtracting the lumen, lignin, and hemicellulose areas from the RVE. The FE models corresponding to HS-1 and HS-6 are presented in Figure 5.

Using the geometrical data listed in Table 4 and the properties of the constituents (Table 3), a FE model is generated for all the considered structures (as given in Table 2) to estimate the elastic properties of JF under all possible loading applications [38].

The possible loading cases are longitudinal loading, in-plane transverse loading, out-of-plane transverse loading, in-plane shear, and out-of-plane shear loading. A solid 186 element has been used to describe the model generated for the analysis. This solid 186 is defined by 20 nodes, and each node possesses three directional freedoms, i.e., in the X, Y, and Z directions [41]. Converged FE models are used for the analysis. One-eighth of the RVE is modelled for the analysis in terms of symmetry from the perspectives of loading, geometry, and boundary conditions.

Before finding the required properties of the FE model, the model needs to ensure that the selected unit cell should reflect the total behavior of the selected material. For that, the nodes corresponding to the X = 0, Y = 0, and Z = 0 areas are arrested to move in the X, Y, and Z directions, respectively. Multipoint constraints are applied to the corresponding nodes of the FE model in the positive directions [41,42]. The longitudinal modulus is obtained by applying uniform pressure parallel to the fiber (Z axis) and, using Hooks’ law, the longitudinal modulus is obtained (Figure 3a). The transverse modulus is obtained by applying load in the X and Y directions of the FE model, respectively (Figure 3b,c). The in plane shear modulus is calculated using models loaded in the XZ plane, and the out-of-plane modulus is obtained by applying load in the XY plane. The major Poisson’s ratio is calculated by dividing −ε_2_/ε_1_ where ε_1_ is the longitudinal strain ε_2_ is the lateral strain of the composite material.

### 4.2. Second Stage of Homogenization

The final JFR composites are evaluated by considering the JF properties, which are obtained by using the methodology proposed in Section 4.1. Considering six hierarchical structural models and their properties, the final JFR composites are estimated and presented in Section 5.2. The concentration of lumen percentage differs between the six HSs.

Using the properties of JF from the first stage of homogenization, the fiber-reinforced polypropylene composite is estimated. The second stage of homogenization is used to Figure out how different HS structures affect the final properties of the composite.

### 4.3. Validation of Simulation Studies

The FE models are validated by comparing the results with experimental results [43,44]. The experimental results are available for 10 and 30% volume fraction (12.95% and 36.45% weight fraction). Using the method proposed in Section 3 and Section 4, the longitudinal modulus is predicted and compared to the experimental and analytical results (Table 5).

## 5. Results and Discussions

### 5.1. Simulation Results of JF Using Micromechanics Approach (First Stage of Homogenization)

**Figure 6:**
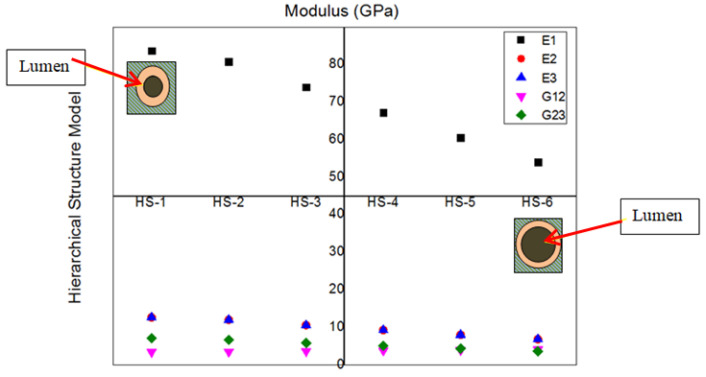
Modulus for different Hierarchical models. (Lumen is shown with red arrows).

Figure 6 shows the variation of fiber directional or longitudinal modulus (E_1_), transverse modulus (E_2_ and E_3_), in-plane (G_12_) and out-of-plane shear modulus (G_23_). Among all the moduli, E_1_ is more than E_2_ or E_3_, G_12_, and G_23_. A declining trend is observed in all the moduli except G_12_ from HS-1 to HS-6. Changing the HS from HS-1 to HS-6 decreases the E_1_ from 80.78 to 54.12 Gpa. The possible reason for the decrease is the increase of lumen (hollowness) in the fiber. Increasing the lumen decreases the cellulose percentage, and cellulose is the main load-bearing element of the fiber [29]. About 33% of E_1_ is decreased by changing the HS from HS-1 to HS-6. The reason for the decrement is an increase in the lumen percentage from HS-1 to HS-6. Compared to E_1_, the transverse modulus of E_2_ and E_3_ is affected more due to lumen percentage. As a result, 42.78% of E_2_ decreased from HS-1 to HS-6. A different scenario is observed in shear modulus. The in-plane shear modulus is increases from HS-1 to HS-6, which means that the lumen percentage is not affected by the in-plane modulus and the contribution of cellulose is dominated by the decrease caused by the lumen. As a result, the G_12_ improves by 44.4% from HS-1 to HS-6 models. G_23_ become less bright as the lumen percentage goes up, just as lumen does with E_1_ and E_2_.

The ratio of lateral strain to the longitudinal strain of the composite material gives the Poisson’s ratio. Composite materials have two types of Poisson’s ratios. Major Poisson’s ratio (ν_12_ or ν_13_) minor Poisson’s ratio (ν_23_ and ν_21_). For transversely isotropic materials such as (E_2_ = E_3_), the magnitude of the major Poisson’s ratio ν_12_ or ν_13_ is the same and minor Poisson’s ratio ν_21_ and ν_23_ are same. However, the presence of lumen inside the JF makes the difference between the major Poisson’s ratio ν_12_ and ν_13_ and the minor Poisson’s ratio ν_21_ and ν_23_.

**Figure 7:**
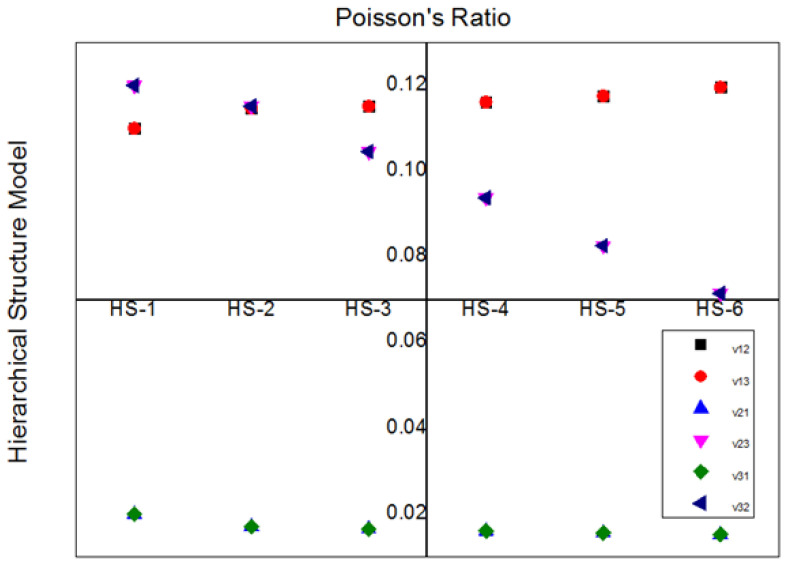
Poisson’s ratio for different Hierarchical Models.

The major Poisson’s ratios ν_12_ and ν_13_ increase from HS-1 to HS-6 due to an increase in lumen percentage (Figure 7). This response is caused by excessive deformation in longitudinal loading due to lumen in the transverse loading. The minor Poisson’s ratios ν_23_, ν_32_ decrease from HS-1 to HS-6. In the transverse loading, excessive deformation in the longitudinal loading due to lumen is the reason for this response. Ν_31_ and ν_32_ are the same, and the magnitude is very small, and the changes in these properties are constant from HS-1 to HS-6. From the whole of Figure 7, it is observed that the ν_12_, ν_13_ magnitudes are the same. Moreover, the ν_23_ and ν_32_ magnitudes are also the same.

**Figure 8:**
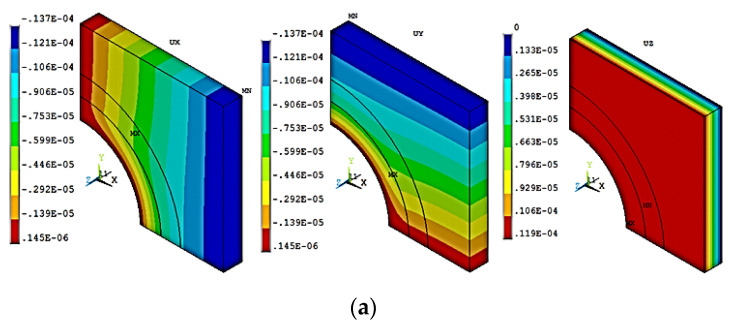

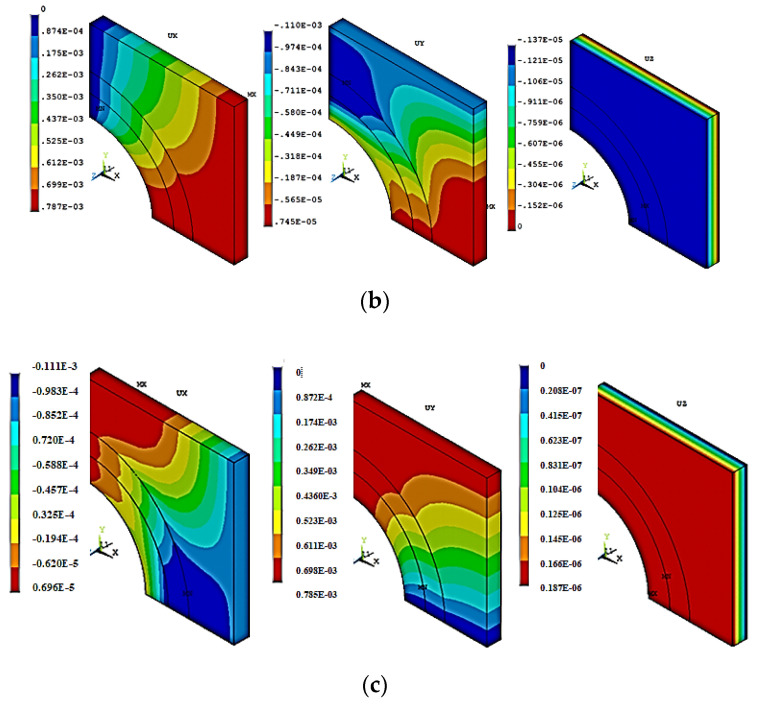
Finite Element contours under longitudinal (**a**), transverse (2-direction) (**b**) and transverse (3-direction) (**c**) loadings of HS-1 model in X, Y and Z directions.

Figure 8 depicts the deformation contours of the FE model under longitudinal and in-plane and out-of-plane transverse loads. The deformation contours of the HS-1 model in the X (ux), Y (uy), and Z (uz) directions under the longitudinal loading direction are shown in Figure 8a [42]. The lumen behavior differs for the HS finite element model when subjected to longitudinal, in-plane transverse, and out-of-plane transverse directions.

Figure 8b,c show the FE deformation contours in X, Y, and Z directions for the FE model under in-plane transverse and out-of-plane transverse loading (X and Y-directions). The JF with lumen behaved differently in the in-plane transverse directions than a transverse isotropic material (Figure 8b). Figure 8a shows the FE contours of the HS-1 model subjected to directional fiber loading.

### 5.2. Simulation Results of JFR Polypropylene Using Micromechanics Approach (Second Stage of Homogenization)

**Figure 9:**
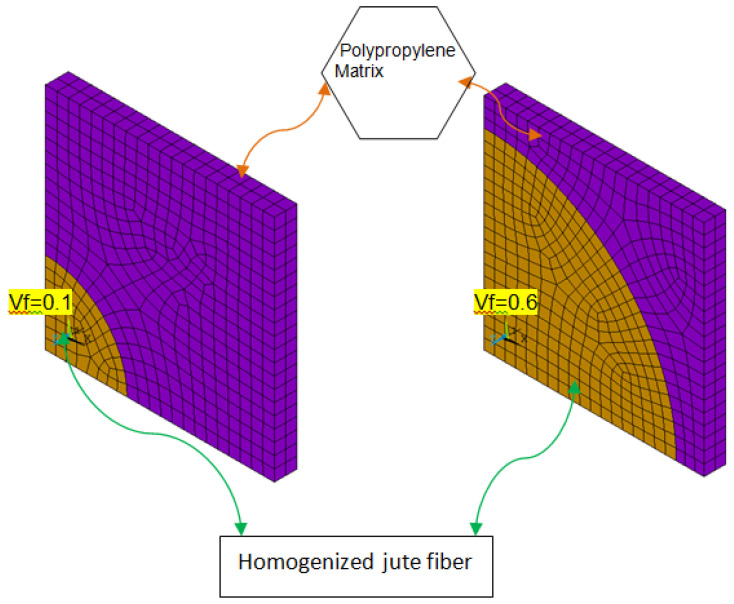
Finite element models of jute fiber reinforced polypropylene.

In this section, the elastic properties of JFR polymer composites are presented by conducting analytical and simulating studies. Jute, a NF with lumen, lignin, hemicellulose, and cellulose was considered for the study. The homogenized properties of JF with different HS are measured. Six HSs models were considered, and each structure is different based on the lumen percentage. The homogenized properties of six hierarchically structured JFs were further used in the second level of homogenization to quantify the JFR polymer matrix composite. Figure 9 depicts the FE models at 0.1 and 0.6 volume fractions, as well as the homogenized JF and polypropylene matrix representations.

**Figure 10:**
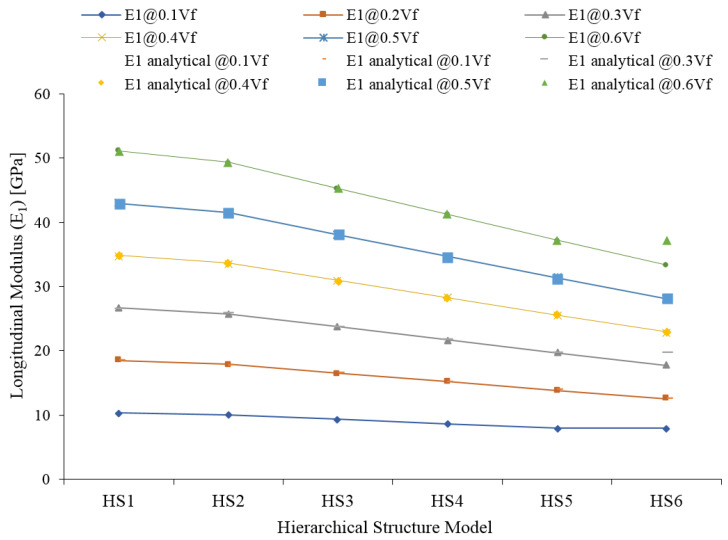
Longitudinal modulus (E_1_) of jute fiber reinforced composite.

The longitudinal modulus (E_1_) of the JFR polymer composite is presented in Figure 10. The longitudinal modulus decreases at all volume fractions of JF in all Hs models considered for the study, from HS-1 to HS-6. The decrease in E_1_ is greater at higher volume fractions than at lower volume fractions of JF. Lumen is the primary parameter that determines the property of the HS model. The modulus E_1_ decreases as the lumen percentage increases from HS-1 to HS-6. Analytical results are also compared with numerical results and good agreement is found. In the authors' previous studies, it was found that the synthetic fiber reinforced composite longitudinal modulus (E_1_) is not affected by deboning defects and moisture defects [24,41], but the behavior of the NF is different when compared to natural fiber. The HS of the NF has a considerable influence on the E_1_. From HS-1 to HS-6, the percentage of lumen has increased as a result; the cellulose percentage has decreased as a result, the E_1_ has decreased from HS-1 to HS-6. Not all the JFs will show the same HS, and selecting the fibers with a high cellulose percentage or low lumen percentage is desirable to achieve the high longitudinal modulus. Perfect alignments between the analytical and FE results are observed at every volume fraction of JF.

**Figure 11:**
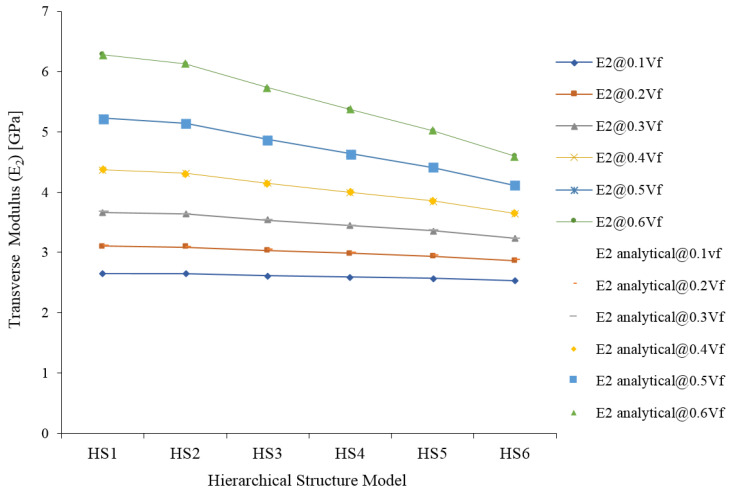
Transverse Modulus (E_2_) of jute fiber reinforced composite.

Transverse Modulus (E_2_) also decreased from HS-1 to HS-6 and at a higher volume fraction of JF; the property loss is high, whereas at lower volume fraction of JF the effect of HS is negligible (Figure 11). The main changing parameter of HS is the percentage of lumen in the structure, it increases from HS-1 to HS-6, and the increase of the percentage of lumen decreases the contribution of cellulose in the fiber as a result of the decrease of the modulus. The simulation results are compared with the analytical results, and both results are in good agreement.

**Figure 12:**
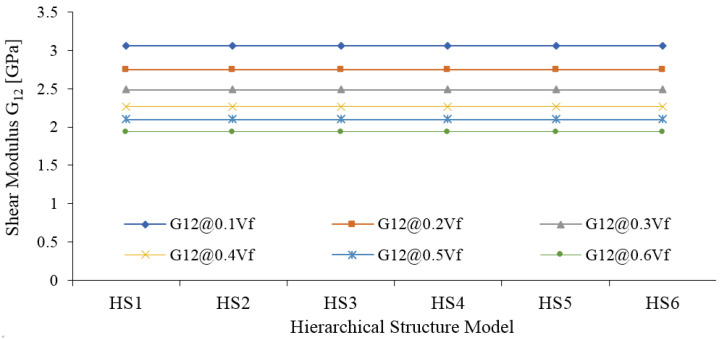
In-plane shear modulus (G_12_) of Jute fiber composites.

The in-plane shear modulus (G_12_) is not changed with HS (Figure 12). The magnitude of G_12_ magnitude increases with increasing fiber percentage; however, the influence of HS is negligible on this property. This means that increasing lumen percentage or decreasing cellulose is not affected by final G_12_.

**Figure 13:**
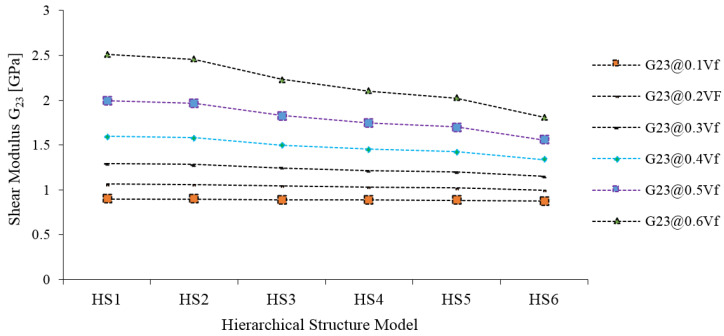
Out-of-plane shear modulus (G_23_) of Jute fiber composites.

The out-of-plane shear modulus G_23_ is influenced by the type of HS of JF, especially at volume fractions of 0.6, 0.5, 0.4, and 0.3 of JF (Figure 13). At lower volume fractions of JF, i.e., 0.1 and 0.2, no such changes are observed in the G_23_.

**Figure 14:**
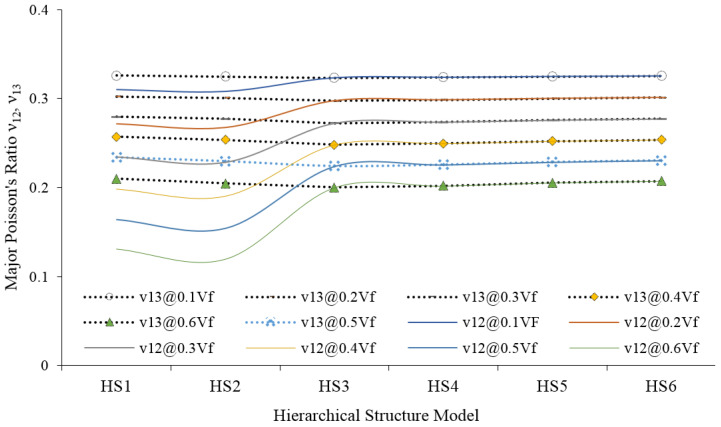
The major Poisson’s ration (ν_12_ and ν_13_) of jute fiber reinforced composites.

Figure 14 shows the major Poisson’s ratios ν_12_ and ν_13_ of a JFR polymer composite. ν_12_ is estimated from the transverse and longitudinal strain of the RVE. The longitudinal strain is obtained by applying the load in the direction (1) of the fiber, under the same load, the RVE will experience transverse strain (ε_2_), and then the ratio of the transverse strain (ε_2_) to the longitudinal strain (ε_1_) will be the major Poisson’s ratio (ν_12_). Similarly, ν_13_ is obtained by dividing (ε_3_) and (ε_1_) of the JFR composite of the same RVE.

Compared with synthetic fiber composites, ν_12_ and ν_13_ are not the same for HS-1, HS-2 and HS-3 structured composites. The highest percentage of cellulose is responsible for this deviation in HS-1, HS-2 and HS-3 model structures. The largest lumen content giving the same response in transverse directions (2 and 3). Unlike ν_12_ and ν_13_ of JF, the major Poisson’s ratio of JFR polypropylene composite (Figure 7) shows a clear variation up to HS-3; after that, the ν_12_ becomes the same as the ν_13_. This behavior is only due to the matrix phase.

**Figure 15:**
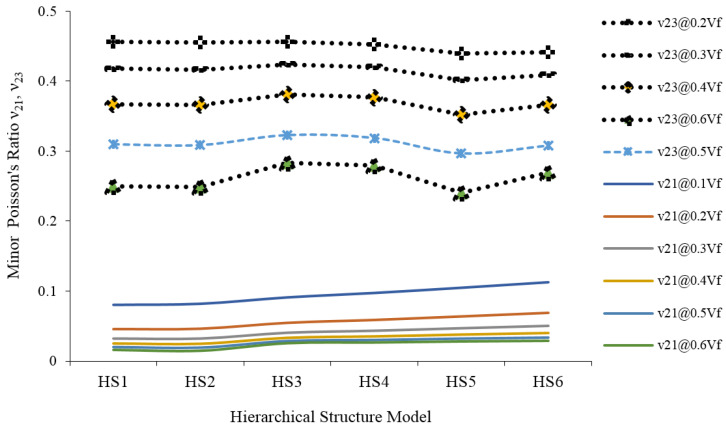
The minor Poisson’s ration (ν _21_ and ν _23_) of jute fiber reinforced composites.

The minor Poisson’s ratio ν21 and ν23 are presented in Figure 15. Compared to ν_21_, the magnitude of ν_23_ is very high. The reason for the high magnitude is the increased response of the lumen of the JF.

Currently, jute fiber is utilized in a variety of industries, such as textiles, vehicles, and even some load-bearing applications. In the automotive industry, bio-polymers and advanced composites made from jute are used for manufacturing parts like cup holders, trunk liners, and door panels [45,46,47].

## 6. Conclusions

Natural fiber reinforced composites manufactured with jute fibers (JFs) and polypropylene matrix are analyzed considering the hierarchical structure (HS) of jute. Different HSs are considered based on different percentages of JF constituents, such as cellulose, lignin, hemicellulose, and lumen.

The type of HS of JF was found to influence the elastic properties of jute fiber reinforced (JFR) polyester composites. Analytical and micromechanics-based finite element models are used to generate the results. A perfect alignment is observed between the analytical and simulation results.

Increasing the lumen percentage from 13 to 35% in the JF from HS-1 to HS-6, decreases the longitudinal modulus (E_1_) of the JF from 83.75 to 54.12 GPa. The transverse modulus (E_2_ and E_3_) of the same fiber decreased from 12.70 to 6.90 GPa. The in-plane shear modulus G_12_ increased from 3.46 to 4.19 GPa, while the out-of-plane shear modulus G_23_ decreased again from 7.12 to 3.69 GPa. The lumen and cellulose percentages of JF are not significantly influenced by E_1_ and E_2_ and the out-of-plane shear modulus (G_23_) at a lower volume fraction of JF in the polypropylene matrix. From both analytical and numerical simulation models, it is found that the influence of HS on in-plane shear modulus is negligible.

The major Poisson’s ratio (ν_12_ and ν_13_) of JF for different HS models is the same. However, for JFR polyester composites, there was a clear difference in ν_12_ and ν_13_ for HS-1, HS-2, and HS-3 models. This is only due to the role of the polypropylene matrix. For JF with different HS models, the magnitude of minor Poisson’s ratio ν_21_ is much smaller than ν_23_. The same trend continued for JFR composites due to more elongation of the lumen under transverse loading.

## Figures and Tables

**Table 1:** Longitudinal modulus from experimental studies.

Weight Fraction of Jute Fiber (%)	Young’s Modulus in GPa						
	Specimen 1	Specimen 2	Specimen 3	Specimen 4	Specimen 5	Mean	SD
12.95	12.648	10.113	15.698	15.169	15.169	13.759	1.055
36.47	22.755	19.698	20.226	25.283	18.689	21.330	1.194

**Table 2:** Volume fraction of fiber constituents with different Hierarchical Structure.

Model	Volume Fraction of Cellulose V_c_ (%)	Volume Fraction of Hemicelluloses V_hc_ (%)	Hierarchical Structure Volume Fraction of lignin V_l_ (%)	Volume Fraction of Lumen V_lm_ (%)
HS-1	61	14	12	13
HS-2	59	14	12	15
HS-3	54	14	12	20
HS-4	49	14	12	25
HS-5	44	14	12	30
HS-6	39	14	12	35

**Table 3:** Constituent properties of Jute Fiber [36].

Constituent	E_1_ [Gpa]	E_2_ [Gpa]	E_2_ = E_3_ [Gpa]	G_12_ [Gpa]	ν_12_ [–]
Cellulose	134	27.2	27.2	4.4	0.10
Hemicellulose	8	4.0	4.0	2.0	0.20
Lignin	4	4.0	4.0	1.5	0.33

**Table 4:** Geometrical details of the Hierarchical Structure for FE Models.

Constituent	Model											
	HS-1		HS-2		HS-3		HS-4		HS-5		HS-6	
	r_i_ (nm)	r_o_ (nm)	r_i_ (nm)	r_o_ (nm)	r_i_ (nm)	r_o_ (nm)	r_i_ (nm)	r_o_ (nm)	r_i_ (nm)	r_o_ (nm)	r_i_ (nm)	r_o_ (nm)
Lumen	2.03	2.03	2.18	2.18	2.52	2.52	2.82	2.82	3.09	3.09	3.33	3.335
Lignin	2.03	2.82	2.18	2.93	2.52	3.19	2.82	3.43	3.09	3.65	3.33	3.865
Hemicellulose	2.82	3.51	2.93	3.61	3.19	3.82	3.43	4.02	3.65	4.22	3.86	4.4
Cellulose area [nm^2^]	61.295		59.058		54.036		49.028		44.013		39.178	

**Table 5:** Comparison of longitudinal modulus from experimental and simulation studies.

Weight Fraction of Jute Fiber (%)	Young’s Modulus in Gpa			% Error of FEM Results with Experimental Results
	Experimental	Analytical	FEM	
12.95	13.759	13.88	13.98	1.64%
36.47	21.330	21.42	22.62	6.04%

## Data Availability

Not applicable.

## Nomenclature

FΦ	Force on the RVE
Fα	Force taken by Lumen
Fβ	Force taken by lignin
Fγ	Force taken by hemi cellulose
Fδ	Force taken by cellulose
Fm	Force taken by the Matrix
σΦ	RVE stress under longitudinal loading
σα	Lumen stress under longitudinal loading
σβ	Lignin stress under longitudinal loading
σγ	Hemi cellulose stress under longitudinal loading
σδ	Cellulose stress under longitudinal loading
AΦ	cross-sectional area of the composite
Aα	cross-sectional area of the Lumen
Aβ	cross-sectional area of the lignin
Aγ	cross-sectional area of the hemi cellulose
Aδ	cross-sectional area of the cellulose
Am	cross-sectional area of the Matrix
σΦ	RVE stress under longitudinal loading
σα	Lumen stress under longitudinal loading
σβ	Lignin stress under longitudinal loading
σγ	Hemi cellulose stress under longitudinal loading
σδ	Cellulose stress under longitudinal loading
E_1_Φ	Longitudinal Elastic modulus of RVE
E_1_α	Elastic modulus of Lumen
E_1_β	Elastic modulus of Lignin
E_1_γ	Elastic modulus of Hemi cellulose
E_1_δ	Elastic modulus of Cellulose
HS	Hierarchical Structures
JF	Jute Fiber
JFR	Jute Fiber Reinforced
NF	Natural Fiber
NFR	Natural Fiber Reinforced
RVE	Representative Volume Element
ε_1_Φ	Longitudinal Strain of RVE
ε_1_α	Longitudinal strain of Lumen
ΔΦ	Transverse deformation of RVE
Δα	Transverse deformation of Lumen
Δβ	Transverse deformation of Lignin
Δγ	Transverse deformation of Hemi cellulose
Δδ	Transverse deformation of Cellulose
Δm	Transverse deformation of matrix
ε_2_Φ	Transverse Strain of RVE
ε_2_α	Transverse strain of Lumen
ε_2_β	Transverse strain of Lignin
ε_2_γ	Transverse strain of Hemi cellulose
ε_2_δ	Transverse strain of Cellulose
ε_2_m	Transverse strain of matrix
ε_2_Φ	Transverse Strain of RVE
